# Neuroimmunology and Novel Methods of Treatment for Acute Transverse Myelitis

**DOI:** 10.7759/cureus.17043

**Published:** 2021-08-09

**Authors:** Brian Fiani, Claudia Covarrubias, Ryan Jarrah

**Affiliations:** 1 Neurosurgery, Desert Regional Medical Center, Palm Springs, USA; 2 School of Medicine, Universidad Anáhuac Querétaro, Santiago de Querétaro, MEX; 3 College of Arts and Sciences, University of Michigan - Flint, Flint, USA

**Keywords:** neuroimmunology, plasma exchange therapy, immunomodulatory therapy, fampridine, immunosuppressive agents, restricted progenitor cells

## Abstract

Acute transverse myelitis (ATM) is a rare, immune-mediated pathology that is defined as an adverse inflammatory response in the spinal cord leading to neurologic injury. The pathophysiology of ATM is poorly understood, with no apparent differences in age, ethnicities, or race, along with variable radiographic and clinical presentation. Therefore, in this review, we will characterize what is known about ATM’s etiology and diagnostic criteria, and relate it to properties of neuroimmunology. Moreover, we will further discuss current treatment options, along with potential novel methods, to provide a comprehensive overview of the status of ATM’s research development. Among these novel treatments, potassium blockers reveal exciting early outcomes in restoring neurologic motor function. In addition, human glial progenitor cell transportations have been described as a potential treatment through integrating and remyelinating lesion sites. Nevertheless, despite these novel methods, there is a paucity of clinical trials establishing ATM’s immunopathology and the therapeutic role of potential treatment methods. Therefore, we will highlight the importance of larger well-designed clinical trials in revealing significant biomarkers of injury and recovery.

## Introduction and background

Acute transverse myelitis (ATM), first described in 1882, is a rare, immune-mediated inflammation of the spinal cord affecting people of any age, race, or gender [1, 2]. This demyelinating disorder encompasses a heterogeneous group of inflammatory disorders that can present as an acute and sub-acute dysfunction of the spinal cord, with a bimodal peak incidence among the ages of 10-19 years and 30-29 years, respectively [2]. Herein, we describe the neuroimmunology of ATM along with the immunopathogenesis and the traditional treatment methods in order to set the foundation for describing the novel treatment methods from recent clinical trials. The incidence of ATM is estimated to be between 1 and 8 new cases per 1 million people per year, with no apparent differences between ethnicities or races. Furthermore, it is estimated that approximately 25% of the cases occur in children alone [2, 3]. Clinically, it affects sensory, motor, and autonomic systems causing neurological deficit and long-term disability in up to one-third of the affected patients [3, 4]. Clinical signs of ATM are propagated by an interruption in the ascending and descending neuroanatomical pathways located in the transverse plane of the spinal cord, causing a rapidly progressing muscle weakness or paralysis that initiates in lower extremities which then spreads to upper extremities, dependent on affected spinal cord level, at varying degrees of severity [2, 4]. Additionally, patients can present with diminished pain, temperature, and vibratory sensations [2]. Neuropathic pain may occur in the midline or in a dermatomal distribution that can be correlated to the anatomical level of the lesion [1, 4].

**Figure 1:**
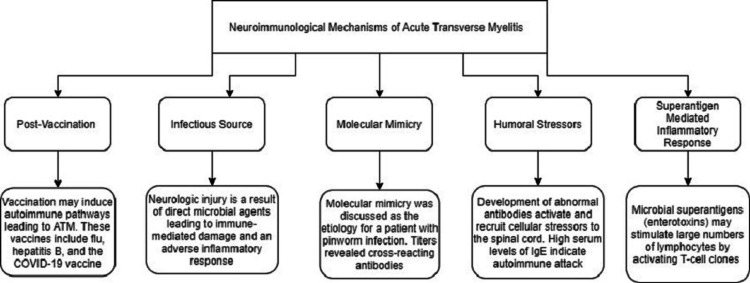
Current theories of the neuropathogenesis of ATM. ATM: acute transverse myelitis; COVID 19: coronavirus disease 2019

Although there are no exact identifiable etiologies of ATM, there have been a number of attributed conditions that are broadly divided into: post-infectious, systemic inflammation, multifocal central nervous system diseases, and idiopathic conditions (Table 1) [1]. Once the setting of another illness is ruled out, idiopathic ATM accounts for approximately 15-30% of the cases and is usually associated with a monophasic syndrome with low risk of relapse [2-4]. To date, there are neither familial risk factors nor recognized genetic contributions linked to the disease [5]. Furthermore, 10-30% of patients with ATM go on to develop multiple sclerosis (MS) over a five to ten-year period, making it a presenting feature of MS [2]. Most recently, there have been multiple case reports associating both coronavirus disease 2019 (COVID-19) infection and COVID-19 vaccination to the development of ATM [6, 7]. Other potential propagators of myelitis not mentioned in Table 1 include myelitis associated with the oncological treatment of immune checkpoint inhibitors (ICIs) [8, 9]. Figure 1 highlights theories of immunopathogenesis that manifest ATM [10].

**Table 1:** Identifiable causes and generalized diagnostic workup for acute transverse myelitis. CNS: central nervous system, MRI: magnetic resonance imaging, CSF: cerebrospinal fluid, PCR: polymerase chain reaction, NMO: neuromyelitis optica; PET: positron emission tomography; SARS CoV-2: severe acute respiratory syndrome coronavirus 2. Most recently *COVID-19 vaccine ChADOx1nCoV-19 (AZD1222)

Disorders		Diagnostic Tests
Immune system disorders	Aquaporin-4 autoantibody, acquired CNS demyelinating disease (multiple sclerosis, neuromyelitis optica), post-infectious or post-vaccine autoimmune phenomenon, paraneoplastic immune response, other antibody-mediated conditions; post-vaccination*	Clinical examination, serologic studies, chest and joint radiography; brain MRI with gadolinium enhancement, CSF examination for cell count and differential count, oligoclonal bands, and IgG index, tests of visual evoked potentials, serum NMO-IgG testing; chest radiography, CT scan, PET scan, CSF paraneoplastic antibody panel, comprehensive serum; recent history of vaccination
Viral infections	Herpesviruses, *Cytomegalovirus*, Epstein-Barr, *Flaviviruses*, echovirus, hepatitis B, mumps, measles, rubella, SARS-CoV-2	History of recent infection, blood serologic studies, CSF culture, PCR. Imaging as indicated
Bacterial infections	Syphilis, middle-ear infections, *Campylobacter jejuni* gastroenteritis, *Mycoplasma* bacterial pneumonia	
Fungal infections	*Aspergillus,* *Blastomyces*, *Coccidioides*, *Cryptococcus*	
Parasitosis	Toxoplasmosis, cysticercosis, schistosomiasis, angiostrongyliasis	
Vascular disorders	Arterial-venous fistula; dural arterial-venous fistula; intraspinal cavernous malformations; fibrocartilaginous embolism	Angiogram, MRI, and other imaging as indicated
Other inflammatory disorders	Sarcoidosis, systemic lupus erythematosus, Sjogren’s syndrome, mixed connective tissue disease, scleroderma; Bechet’s syndrome	Medical history and physical exam; autoantibodies; labial gland/skin biopsy, pathergy test. Imaging as indicated

In 2002, the Transverse Myelitis Consortium Working Group delineated diagnostic criteria for idiopathic and disease-associated ATM [11, 12]. Diagnosis is based on a thorough medical history, complete neurological examination, and radiological findings [1-3]. The proposed diagnostic criteria consist of three main elements: neurological dysfunction attributable to the spinal cord, T2 hyperintense signal change on MRI, and absence of compressive cord lesion [12]. MRI with gadolinium enhancement detecting inflammatory spinal cord lesions, more commonly found at the thoracic level, can confirm the clinical syndromic diagnosis of myelopathy [2-4]. However, full-axis MRI can also be used to assess differential diagnoses and help rule out underlying myelopathy-causative conditions, including but not limited to MS, spinal cord abscesses or masses, vertebral body compression fractures, and spondylitis [3, 4]. Other diagnostic criteria include blood exams that help detect infections, vitamin deficiencies, and presence of autoantibodies including anti-aquaporin-4 (APQ-4)-IgG autoantibodies, anti-myelin oligodendrocyte glycoprotein (MOG) autoantibodies, antinuclear antibodies (ANA), Ro/SSA, La/SSB, and HIV autoantibodies. A lumbar puncture (LP) and cerebral spinal fluid (CSF) analysis can also help identify an increase in number of proteins and white blood cells (WBCs) [1, 12].

Supplementary diagnostic tools can also include urodynamic studies, electrodiagnostic testing through nerve conduction velocity, and ophthalmological examination [13]. Additionally, a current and undergoing clinical trial, injured spinal cord pressure evaluation study-TM (ISCoPE-TM), proposes a novel diagnostic method consisting of an intraspinal pressure (ISP) and spinal cord metabolites monitoring devices in order to assess its feasibility and safety in patients with ATM [14]. This study, being the first of its kind, will help determine whether a swollen and enlarged spinal cord in patients with ATM raises ISP and consequently causes deranged and measurable spinal cord metabolites such as glucose, lactate, pyruvate, glycerol, and glutamate [14].

## Review

Treatment: Traditional methods

There is currently no effective cure for ATM, and available therapies aim to alleviate symptoms by attenuating spinal cord inflammation as well as immune-mediated destruction of myelin [3]. If the acute phase of ATM is detected, initial immunotherapy aims to cease disease progression [4]. First-line standard anti-inflammatory treatments include high-dose IV corticosteroids such as methylprednisolone or dexamethasone. Additionally, second-line treatments include plasma exchange (PLEX) and IV immunoglobulin (IVIG), which are reserved as rescue therapies for steroid-unresponsive patients [3, 4, 15]. A retrospective study published in 2009 aimed to study the outcome of PLEX-treated versus steroid-only-treated attacks in relapsing extensive ATM appearing to be a safe add-on therapy maximizing improvement rates [16]. An additional study published in 2016 concluded that monophasic ATM relapse was more likely to improve after PLEX+ high-dose IV methylprednisolone (IVMP) when compared to IVMP alone [17]. However, a multi-center randomized controlled trial of IVIG compared with standard therapy for the treatment of ATM in both adult and pediatric populations published in 2017 was unable to determine treatment effect due to the limited sample size obtained [15]. The benefits of rescue therapies remain to be established as their therapeutic effects still remain controversial. In the presence of chronic disease or first and second-line treatment-resistance, other immunomodulatory therapies such as cyclophosphamide, mycophenolate, azathioprine, or rituximab have been considered for maintenance treatment in neuromyelitis optica spectrum disorder (NMOSD). Well-designed clinical trials specific to ATM are necessitated in order to establish long-term benefits and side effects of immunosuppressive agents [9, 18].

Treatment: Novel methods

There are several investigational therapies that have undergone or are currently undergoing clinical trials. The potassium channel blocker fampridine, also known as dalfampridine or 4-aminopyridine, has been studied since the 1970s due to its effects in potentiating neurotransmitter release in muscles, increasing post-synaptic action potentials in the spinal cord [5]. Although most clinical trials have been conducted on MS, a study published in 2017 evaluated the efficacy of the extended-release formulation of dalfampridine (D-ER) in idiopathic ATM showing a trend in walking-speed improvement in 85% of the study arm compared to 69% in placebo group, respectively [5].

In 2012, Walczak et al. published the first study evaluating the therapeutic potential of human glial restricted progenitor cells (hGRPs) in an adult rat model of focal inflammatory demyelination, such as occurs in transverse myelitis [19]. Additionally, the safety and tolerability of transplantation in subjects with ATM is currently being studied by Q Therapeutics, Inc. in an ongoing clinical trial. It has been postulated that these cells, once transplanted into the spinal cord-demyelinated lesions, will integrate and remyelinate target areas as well as provide trophic support for damaged axons [20]. Lastly, a case report published in 2012 proposed microsurgical nerve transfer as a therapeutic option for ATM aiming to restore function to affected nerves by transferring axons from a functioning donor nerve. This single case report concluded that the proposed surgical treatment could be utilized for cases resulting in permanent deficits from TM [21]. Overall, larger well-designed studies are warranted in order to further examine the effects of these novel treatments and to determine their therapeutic role.

Limitations and clinical trial importance

Clinical trials that analyze the immunological factors that contribute to ATM, along with defining therapeutic roles of novel treatments are space. Nevertheless, the development of these clinical trials is crucial in the pursuit of improving the general and clinical understanding of ATM’s pathogenesis and developing therapeutic models. The pathophysiology of ATM remains to be poorly understood, therefore, the molecular signaling pathways that manifest into ATM’s clinical presentation are unclear [22]. This is further complicated by the fact that ATM’s etiologies are not established, as casual relationships between different sources have not been widely accepted or proven [23]. Studies that can determine the autoimmune or immunopathogenic mechanisms of ATM would provide a heightened determination of crucial biomarkers that signify ATM’s manifestations. To date, several autoimmune antibodies such as NMO-IgG and MOG-IgG have been proposed as probably biomarkers, without being widely integrated into the workup for ATM’s prognosis [24]. Further studies on ATM’s immunopathology would also allow for the identification of inflammatory triggers on the spinal cord, increase the knowledge of the cellular and humoral factors that induce such an injury, while also potentially identifying modalities to the inflammatory response [10]. Therefore, clinical trials supporting the characterization of this rare pathology will clearly allow for an augmented clinical response and better patient outcomes [3].

In addition, one of the leading limitations in ATM’s diagnostic workup is its associations and similarity to related autoimmune disorders. These disorders include Sjoren’s syndrome, systemic lupus erythematosus (SLE), multiple sclerosis (MS), acute disseminated encephalomyelitis, and neuromyelitis optica [25]. The development of further clinical trials would allow for differentiation and determination of the relationship ATM has with these other conditions. In addition, theories have been proposed to indicate that ATM may have vascular, oncological, bacterial, and viral sources, such as its relation to the COVID-19 pandemic [6]. Therefore, it is conceivable that multiple mechanisms may be involved in ATM’s presentation, with theories such as molecular mimicry being among the probable theories behind an infectious source [10]. Nevertheless, clinical trials focused on ATM are uncommon, with the characterized trials being summarized in Table 2. Further studies utilizing nerve conduction assessments and MRIs would ideally provide prognostic value in uncovering the mysteries of this condition [26]. Moreover, longitudinal and larger studies would allow for enhanced diagnostic criteria and formulation of a comprehensive patient and treatment profile.

**Table 2:** Clinical trials assessing therapeutic role of experimental ATM treatment methods ATM: acute transverse myelitis; D-ER: dalfampridine extended-release; SEP: somatosensory evoked potentials; MEP: motor evoked potentials; MS: multiple sclerosis; CMCT: central motor conduction time

Author	Objective/Method	Finding
Bruna et al., 2006 [27]	Review patients with ATM to differentiate them from the patients with multiple sclerosis (MS) based on the criteria provided by the Transverse Myelitis Consortium Working Group. Also evaluate laboratory and clinical biomarkers	Twenty four patients fulfilled the criteria for definite ATM, 21 for possible ATM. Five patients were diagnostically converted to MS admission Rankin score was the only independent prognostic factor found
Misra & Kumar, 1996 [28]	Ten patients were studied and set for clinical, MRI, and neurophysiological evaluation. These included somatosensory evoked potentials (SEP), motor evoked potentials (MEP), and and upper and lower limb concentric needle EMGs	All patients had lower limb weakness, with three also having upper limb weakness. Results showed extensive MRI changes, unreportable MEP, and evidence of denervation in leg muscles indicating poor patient outcomes
Kalita et al., 1999 [29]	Nine patients (age range of 12-42 years old) with ATM were clinically assessed with medial and tibial SEP and central motor conduction time (CMCT) to upper and lower limbs. The clinical and SEP studies were repeated after 7 and 90 days of treatment with the clinical and evoked potential studies were repeated after 7 and 90 days of intravenous methylprednisolone	Patients had varying degrees of leg weakness ranging from 0 to 4 on the Medical Research Council scale. Pinprick and joint position sensations in the lower limb were impaired in all patients CMCT in upper limb was abnormal in two patients, and abnormal in eight patients assessing lower limb. Following treatment, both sensory and motor functions improved, but the improvement was more profound in 90-day assessment
Seze et al., 2005 [30]	Retrospective study on 288 patients analyzing the criteria set by the Transverse Myelitis Consortium Group	Forty five out of 288 patients met the inclusion criteria for idiopathic ATM. Prognosis was highly variable among patients
Murthy et al., 1999 [31]	SEP by paraspinal stimulation were studied in six patients with ATM	Five patients showed slowing of conduction across the involved segments following paraspinal stimulation. One patient’s results were not recordable
Absoud et al., 2017 [15]	Randomized controlled trial evaluating whether early addition treatment with intravenous immunoglobulin is beneficial in TM patients compared with standard therapy with IV steroids	Twenty six participants were screened and two were randomized into the study. With the limited sample size, treatment effect could not be determined
Schwartz et al., 2017 [5]	A randomized, double-blind, placebo-controlled study evaluating the role of dalfampridine extended-release (D-ER) on 16 patients with TM. Participants were randomized to receive 10 mg twice daily of either the D-ER or placebo control for 8 weeks and then crossed over. Patients were evaluated based on a 25-foot walk speed	Thirteen patients completed trial, nine individuals showed an average timed walk that was faster with the D-ER. Only four participants reached statistical significance D-ER may be beneficial for patients with ATM to improve neurologic function

## Conclusions

The neuroimmunology of ATM will remain to be a key area of interest in the pursuit of further characterizing this rare pathology. While there remains to be no cure for ATM, along with a paucity of clinical trials that further describe its etiology and treatment, traditional therapies have been characterized to limit the adverse immune response. Novel therapies such as potassium inhibitors and human glial progenitor cell transportation all indicate therapeutic potential, however, the lack of clinical studies supporting their role limits their acceptance as an official treatment modality. It can be generally concluded that if the pathophysiology of ATM remains to be poorly understood, the development of more novel treatments will continue to be limited. Nevertheless, as the neuroimmunology of ATM begins to unfold, predictors and biomarkers of ATM can be established to further study variable courses of ATM. This will ultimately pave the formation of an algorithm to prognosticating and treating ATM to improve future quality of life for future diagnosed patients.
